# Iron metabolism and resistance to infection by invasive bacteria in the social amoeba *Dictyostelium discoideum*

**DOI:** 10.3389/fcimb.2013.00050

**Published:** 2013-09-19

**Authors:** Salvatore Bozzaro, Simona Buracco, Barbara Peracino

**Affiliations:** Department of Clinical and Biological Sciences, University of TorinoOrbassano, Italy

**Keywords:** *Dictyostelium*, *Legionella*, *Mycobacterium*, Nramp1, Nramp2, iron homeostasis, iron genes, host-pathogen interactions

## Abstract

*Dictyostelium* cells are forest soil amoebae, which feed on bacteria and proliferate as solitary cells until bacteria are consumed. Starvation triggers a change in life style, forcing cells to gather into aggregates to form multicellular organisms capable of cell differentiation and morphogenesis. As a soil amoeba and a phagocyte that grazes on bacteria as the obligate source of food, *Dictyostelium* could be a natural host of pathogenic bacteria. Indeed, many pathogens that occasionally infect humans are hosted for most of their time in protozoa or free-living amoebae, where evolution of their virulence traits occurs. Due to these features and its amenability to genetic manipulation, *Dictyostelium* has become a valuable model organism for studying strategies of both the host to resist infection and the pathogen to escape the defense mechanisms. Similarly to higher eukaryotes, iron homeostasis is crucial for *Dictyostelium* resistance to invasive bacteria. Iron is essential for *Dictyostelium*, as both iron deficiency or overload inhibit cell growth. The *Dictyostelium* genome shares with mammals many genes regulating iron homeostasis. Iron transporters of the Nramp (Slc11A) family are represented with two genes, encoding Nramp1 and Nramp2. Like the mammalian ortholog, Nramp1 is recruited to phagosomes and macropinosomes, whereas Nramp2 is a membrane protein of the contractile vacuole network, which regulates osmolarity. Nramp1 and Nramp2 localization in distinct compartments suggests that both proteins synergistically regulate iron homeostasis. Rather than by absorption via membrane transporters, iron is likely gained by degradation of ingested bacteria and efflux via Nramp1 from phagosomes to the cytosol. *Nramp* gene disruption increases *Dictyostelium* sensitivity to infection, enhancing intracellular growth of *Legionella* or *Mycobacteria*. Generation of mutants in other “iron genes” will help identify genes essential for iron homeostasis and resistance to pathogens.

## Introduction

*Dictyostelium discoideum* is a member of the *Amoebozoa* (Schilde and Schaap, 2013). The cells live as unicellular amoebae in deciduous forest soil, feeding on bacteria that are taken up by phagocytosis and dividing by binary fission. Exhaustion of the food supply triggers a shift from growth to development, resulting in cells gathering by chemotaxis into aggregates of several thousands of cells. The tight aggregates transform into elongated sausage-like multicellular organisms, called slugs, in which cells differentiate into pre-stalk and pre-spore subtypes. After extensive migration, the slug eventually culminates into a fruiting body, consisting of a slender stalk of vacuolated cells bearing on top a ball of fully differentiated spores (Kessin, 2001).

Due to their life cycle, easy handling and genetic tractability, *D. discoideum* (in the followings *Dictyostelium*) has long been a preferred model organism for studying basic processes, such as motility and chemotaxis, cell-cell communication and adhesion, cell differentiation, pattern formation and morphogenesis (Bozzaro, 2013). Their ability to phagocytose has been exploited, in the last decade, to investigate dynamics and regulatory pathways of phagocytosis as well as interactions with an increasing number of clinically relevant bacterial pathogens, including *Legionella pneumophila, Mycobacterium avium* or *marinum*, and *Pseudomonas aeruginosa* (Bozzaro et al., 2008, 2013; Cosson and Soldati, 2008; Clarke, 2010; Bozzaro and Eichinger, 2011; Steinert, 2011). In the light of these recent developments, in this review we will discuss the opportunities offered by *Dictyostelium* in investigating the role of divalent metal homeostasis, with a special emphasis on iron, for cell growth, and defense against pathogenic bacteria.

## *Dictyostelium*: professional phagocyte and pathogen host

Wild type *Dictyostelium* strains are strictly dependent on bacteria for growth, though a few selected laboratory strains are able to grow in liquid axenic media by fluid-phase endocytosis, mostly macropinocytosis (Kessin, 2001; Maniak, 2001). Thousands of prokaryotic species are present in the forest soil; how many of them can serve as food for *Dictyostelium* is unknown, but the cells appear to be rather omnivorous. Soil bacteria which have been isolated in association with wild type strains include close relatives of *Burkholderia xenovorans, Stenotrophomonas maltophilia, Enterobacter sakazakii, Pseudomonas fluorescens*, and *Flavobacterium johnsoniae* (Brock et al., 2011). Under laboratory conditions, the cells are able to graze on a very large variety of Gram-negative and Gram-positive bacteria, including different species of *Enterobacter, Serratia, Salmonella, Yersinia, Proteus, Aeromonas, Alcaligenes, Acinetobacter, Staphylococcus, Listeria*, and *Bacillus* (Depraitere and Darmon, 1978). They are also capable of modulating their response to different types of bacteria by activating specific sets of gene transcripts (Farbrother et al., 2006; Carilla-Latorre et al., 2008; Sillo et al., 2008, 2011). In a recent paper, (Nasser et al., 2013) have studied global transcriptional response of wild type and selected mutant cells to a series of Gram-negative and Gram-positive bacteria, and they could show that cells respond differently to these two large families of bacteria. By analysing the transcriptional response to live, in contrast to dead bacteria, they were also able to identify selective gene pathways needed for defense, rather than growth, on either Gram-negative or Gram-positive bacteria, such as the activation of different sets of lysozymes or a set of glycoproteins apparently required for growth on Gram-positive bacteria.

Phagocytosis, both on agar plates or under shaking in simple salt solution, is very efficient. Only a handful of bacteria strains are not phagocytosed, but very few systematic studies have been published in this regard. *Legionella pneumophila* is taken up by macropinocytosis, thus its uptake by natural wild type strains is negligible (Peracino et al., 2010; Balest et al., 2011). *Bacillus* anthracis was reported not to be phagocytosed (Depraitere and Darmon, 1978), but a recent report described its uptake, though no details were offered on the efficiency of phagocytosis (Nasser et al., 2013). Human pathogenic Escherichia coli strains were found to be graze-resistant when co-cultured with *Dictyostelium* cells at high, but not low, density. Whether this was due to the high bacterial density inhibiting phagocytosis or killing the cells was not assessed, except for one strain (Adiba et al., 2010). A correlation between phagocytosis efficiency and bacterial density or previous growth conditions has been also described for *Salmonella typhimurium* (Sillo et al., 2011), *Aeromonas spp.* (Froquet et al., 2007) and for *Klebsiella pneumoniae* (March et al., 2013). Cells are also able to discriminate between edible and less appetizing bacteria. When co-cultured with *S. typhimurium* and *E. coli*, cells depleted *E. coli* from the medium leaving quite unaltered the *Salmonella* (Sillo et al., 2011). Co-culturing with live Gram-negative bacteria was also shown to prime the cells to grow on dead Gram-positive bacteria that otherwise were not utilized as food source (Nasser et al., 2013).

It is nowadays accepted that free-living amoebae and protozoa, by predating bacteria, can become a driving force for the evolution of pathogenic traits, paradigmatic cases being Acanthamoeba castellanii parasitic interaction with *Legionella pneumophila* (Barker and Brown, 1994; Levin, 1996; Steinert et al., 2000); or the variation of the rfb virulence locus in *Salmonella* enterica mediated by intestinal protozoan predation (Wildschutte et al., 2004). The ability of many pathogens to grow in macrophages and cause human diseases appears thus to be a consequence of their adaptation and survival in the normally hostile amoeboid niche (Steinert et al., 2000; Greub and Raoult, 2004; Casadevall, 2008).

Being a bacterial predator, it would be rather surprising that *Dictyostelium* would be an exception on this regard, though *Dictyostelium* parasites naturally occurring in the wild have not been described, likely due to lack of systematic studies. At least for the fungus *Cryptococcus neoformans* it was, however, shown that passage through *Dictyostelium* cells enhanced its virulence in mice (Steenbergen et al., 2003). Invasive pathogenic microbes which have been shown to be able to grow in *Dictyostelium* cells include, in addition to *Cryptococcus, Legionella* (Haegele et al., 2000; Solomon and Isberg, 2000), *Mycobacteria* (Solomon et al., 2003) and Burkholderia species (Hasselbring et al., 2011). *S. typhimurium co*-cultured under optimal nutrient conditions has been shown to enter wild type cells and kill the cells, but its intracellular replication has been documented in autophagic *Dictyostelium* mutants only (Jia et al., 2009; Sillo et al., 2011). Similarly, *K. pneumoniae* is pathogenic for some *Dictyostelium* mutants, not for the parental wild type strain (Benghezal et al., 2006). Capsulated *Neisseria meningitidis* (Colucci et al., 2008) as well as pathogenic *Escherichia coli* strains (Adiba et al., 2010) have been shown to resist degradation, but their intracellular growth has not been documented.

Since *Dictyostelium* can be easily grown in association with bacteria on agar plates, a plaque assay, and in some cases growth assay under shaking, have been used by several labs for screening microbial virulence genes, using either wild type or in some cases mutant cells. Virulence traits have thus been identified in *E. coli* (Adiba et al., 2010), *Pseudomonas aeruginosa* (Cosson et al., 2002; Pukatzki et al., 2002; Alibaud et al., 2008), *Vibrio cholerae* (Pukatzki et al., 2006; Miyata et al., 2011; Zheng et al., 2011), *Stenotrophomonas aeruginosa* and *S. malthophylia* (Alonso et al., 2004; Adamek et al., 2011), *K. pneumoniae* (Benghezal et al., 2006; Pan et al., 2011; March et al., 2013), *Burkholderia coenocepacia* and *B. pseudomallei* (Aubert et al., 2008; Hasselbring et al., 2011).

In contrast to these bacteria, *Legionella pneumophila, Mycobacterium avium*, or *M. marinum* grow rapidly intracellularly, and their interaction with *Dictyostelium* cells has been extensively studied [for recent reviews see (Bozzaro and Eichinger, 2011; Hilbi et al., 2011; Steinert, 2011)]. *In vivo* imaging of the dynamics of infection, favored by the large array of fluorescent probes against cytoskeletal and organelle proteins, has shown that the process is highly conserved between *Dictyostelium* and macrophages. Both *Legionella* and *Mycobacteria* manipulate the endocytic pathway to hinder fusion of the pathogen-containing phagosome with acidic and lysosomal vesicles, favoring association with other compartments, such as the endoplasmic reticulum (Fajardo et al., 2004; Lu and Clarke, 2005; Ragaz et al., 2008; Peracino et al., 2010) and mitochondria (Francione et al., 2009), generating a replication vacuole. Whereas massive intracellular growth of *Legionella* leads to cell lysis, *M. marinum* has been shown to escape intact cells by a novel non-lytic exocytic mechanism that may be active also in macrophages (Hagedorn et al., 2009). Genomic-wide transcriptional changes during infection and proteomic analysis of the *Legionella*-containing vacuole (LCV) have also helped in characterizing the dynamics of infection (Farbrother et al., 2006; Li et al., 2009; Shevchuk et al., 2009; Urwyler et al., 2009).

Infection assays have been exploited particularly with *Legionella* to identify and/or characterize virulence genes. Thus, it has been shown that *Legionella* mutants defective in sigmaS or its effector LqsR, which regulate expression of several genes involved in virulence, motility and transmission, are defective for growth in *Dictyostelium* and macrophages (Tiaden et al., 2007; Hovel-Miner et al., 2009). The Icm/Dot TFSS (Type Four Secretion System) substrate SdhA appears to hinder cell apoptosis, thus the mutant is defective for growth in macrophages and to a lower extent in *Dictyostelium* (Laguna et al., 2006). Mutants in the Icm/Dot effectors SidJ or SidjA (Liu and Luo, 2007), SidC or SidA (Ragaz et al., 2008) display impaired recruitment of endoplasmic reticulum to the LCV, with differential effects on intracellular growth, which was depressed in macrophages and *Dictyostelium* or *Dictyostelium* only for SidJ or SidjA, respectively, but unaltered in the SidC or SidA mutants. The Icm/Dot TFSS effectors LepA and LepB have been shown to regulate non-lytic exocytosis from the host (Chen et al., 2004), whereas Leg proteins regulate LCV traffic, by disrupting the endocytic vesicle traffic of the host (de Felipe et al., 2008). The envelope-associated protein EnhC was found to be required for bacterial growth in macrophages, but not in *Dictyostelium* (Liu et al., 2008). Among the ICM/Dot IV injected effectors, AnkyrinB (AnkB) was shown to be anchored via farnesylation to the LCV and be required for docking polyubiquinated host proteins, favoring intracellular growth, both in macrophages and in *Dictyostelium* (Al-Quadan and Kwaik, 2011).

From the site of the host, the large number of available *Dictyostelium* mutants has been exploited with *Legionella* or *Mycobacteria* to identify host resistance factors, whose genetic disruption leads to enhanced pathogen proliferation. They include cytoskeletal and signal transduction proteins, transcription factors, autophagy and mitochondrial proteins [for recent reviews see: (Bozzaro and Eichinger, 2011; Steinert, 2011)]. Among the several host cell factors so far identified, the iron transporters of the Nramp family have been investigated in detail.

## *Dictyostelium* nramp iron transporters in bacterial infection

Since the discovery of an allelic form of the gene encoding natural resistance associated membrane protein (Nramp)1, which conferred susceptibility to various intracellular microbes (Vidal et al., 1995), the number of studies on this metal transporter family has increased dramatically (Cellier, 2012). The Nramp family is widely distributed, from bacteria to humans (Courville et al., 2006). In mammals, Nramp1 (Slc11A1) expression is restricted to macrophages, but a second Nramp protein, (Nramp2, Slc11A2, or DMT1), is localized in the plasma membrane of several tissues, and mutations have been linked to severe microcytic anemia and serum and hepatic overload (Courville et al., 2006; Shawki et al., 2012). Eukaryotic Nramp proteins are Fe^2+^ and Mn^2+^, possibly Co^2+^, transporters (Forbes and Gros, 2001; Nevo and Nelson, 2006), whereas the bacterial homologs (MntH subfamily) transports mainly Mn^2+^ (Papp-Wallace and Maguire, 2006; Cellier, 2012).

The *Dictyostelium* genome harbors two genes encoding members of this family, which have been named Nramp1 and Nramp2. Nramp1 is the ortholog of mammalian Nramp1, and like the macrophage counterpart, it is localized exclusively in phagosomes or macropinosomes. The protein is recruited from trans-Golgi to phago- or macropinosomes shortly after their closure, and is then retrieved from the vesicles during their post-lysosomal maturation (Peracino et al., 2006). The *nramp1* gene is expressed during growth, up-regulated upon incubation with bacteria and down-regulated upon starvation. In contrast, the Nramp2 protein is phylogenetically closer to α-proteobacteria MntH and to Nramp proteins from yeast, fungi and protists, and is exclusively localized in the membrane of the contractile vacuole (Peracino et al., 2013). The contractile vacuole is a bladder and tubular network, which in *Dictyostelium*, like other free-living amoebae and protozoa exposed to sudden environmental changes, regulates osmolarity. Under hypotonic conditions, water is pumped into the contractile vacuole, giving rise to large vacuoles that fuse with the plasma membrane, expelling their content. Under hypertonic conditions, bladder and tubules flatten, releasing water in the cytosol (Gerisch et al., 2002; Heuser, 2006).

The contractile vacuole membrane is studded with the V-H^+^ ATPase, which pumps protons inside the lumen (Heuser et al., 1993; Clarke et al., 2002). The V-H^+^ ATPase is also rapidly recruited to phagosomes or macropinosomes, shortly after their engulfment (Clarke et al., 2002; Peracino et al., 2006). Thus, both Nramp1 and Nramp2, though in two different compartments, co-localize with the vacuolar ATPase that can provide the electrogenic potential regulating their transport activity. Though still debated, it is likely that the proton gradient generated by the activity of the V-ATPase favors Nramp-dependent iron transport via a symport rather than antiport mechanism (Forbes and Gros, 2001; Courville et al., 2006; Nevo and Nelson, 2006). Experiments with purified phagosomes from *Dictyostelium* wild type and Nramp1-null cells supported this hypothesis, showing also that the protein was essential for iron transport (Peracino et al., 2006). It is likely that Nramp2 in the contractile vacuole acts similarly to Nramp1, but this has not been proven yet.

Both Nramp1 and Nramp2 are dispensable for phagocytosis or growth on non-pathogenic bacteria, but their disruption enhances intracellular growth of *Legionella pneumophila* and, at least for Nramp1, also *Mycobacterium avium* (Peracino et al., 2006, 2013). Constitutive Nramp1 overexpression depresses *Legionella*, not, however, *Mycobacterium*, growth. Interestingly, endogenous Nramp1 gene expression is down-regulated during *Legionella*, but not *Mycobacterium* infection (Peracino et al., 2006). These differences may be related to differences between both pathogens in the establishment of their replication vacuole. In *Mycobacteria* infection, the replication vacuole transiently recruits the vacuolar ATPase during the first 90 min post-infection, retrieves it between 2 and 12 h, avoiding recruitment of lysosomal enzymes while acquiring markers of a post-lysosomal compartment (Hagedorn and Soldati, 2007). *Legionella* instead forms a replicative vacuole that avoids fusion with acidic vesicles, associates with mitochondria and recruits proteins of the endoplasmic reticulum and other trafficking routes, similarly to what occurs in macrophages (Fajardo et al., 2004; Lu and Clarke, 2005; Francione et al., 2009). Fusion with vesicles decorated with Nramp1 is not inhibited, but recruitment of the V-H^+^ ATPase, and thus vacuole acidification, is delayed of several hours, though eventually occurs after 12–24 h post-infection (Peracino et al., 2010). Down-regulating Nramp1 expression, could thus help maintaining in the long run a replication-friendly vacuole, if acidification would occur.

**Figure 1:**
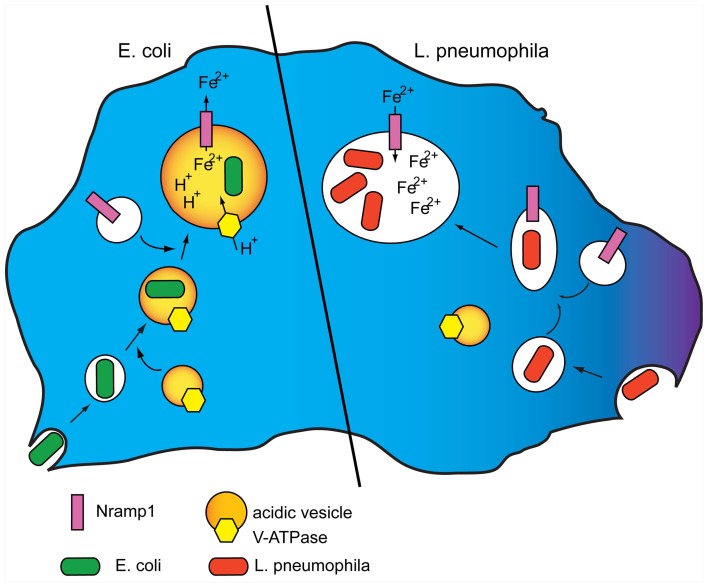
**Model for Nramp1 activity and its manipulation by *Legionella*. (Left)** Nramp1 and the V-H^+^ ATPase are recruited to phagosomes shortly after their uptake. The activity of the vacuolar ATPase generates a proton gradient in the maturing phagosome that provides the electrogenic force necessary for Nramp1 to transport iron, and possibly other divalent metals, to the cytosol, thus depleting the bacteria from an essential nutrient element. **(Right)** *L. pneumophila* is taken up in *Dictyostelium* cells by macropinocytosis. Following uptake, the pathogen inhibits fusion of its vacuole with acidic vesicles bearing the V-H^+^ ATPase. Nramp1 recruitment is not affected, but lack of the electrogenic force neutralizes Nramp1-dependent iron efflux, and can even lead to passive influx of cytosolic labile iron to the advantage of the pathogen. For original data see: Peracino et al. (2010).

Iron is an essential element for virtually all cells, and pathogens such as *Legionella, Mycobacteria*, or *Salmonella* are known to assimilate significant amounts of iron for their metabolism and virulence (Pope et al., 1996; Robey and Cianciotto, 2002; Rodriguez, 2006; Pandey and Rodriguez, 2012; Soldati and Neyrolles, 2012). A systematic analysis of human pathogenic *Escherichia coli* strains showed that *E. coli* genes involved in iron metabolism, such as *irp, fyuA*, and *IroN*, favored resistance to predation by *Dictyostelium* cells (Adiba et al., 2010). Thus, depleting iron from the phagosome via Nramp1 can be an effective host defense strategy to starve the pathogen for iron. Conversely, hindering co-recruitment of the V-H^+^ ATPase by the pathogen, as shown for *Legionella*, not only avoids acidification of the vacuole, thus neutralizing Nramp1-dependent iron efflux, but could even favor iron influx in the vacuole via Nramp1, thus turning Nramp1 to *Legionella* advantage (Peracino et al., 2010) (Figure 1). Experiments with isolated phagosomes showed indeed that inactivating the vacuolar ATPase resulted in passive iron flux (Peracino et al., 2006).

Intracellular growth of *L. pneumophila* is enhanced in Nramp1-null mutants and inhibited in cells overexpressing Nramp1, but the latter effect was found to be reversed by phosphatidylinositide-3 kinase (PI3K) inhibitors or by genetic ablation of PI3K and the phosphatase PTEN (Peracino et al., 2010). The PI(4,5)P2 or PI(3,4,5)P3 phosphatase Dd5P4 (OCRL1) also enhanced *Legionella* growth (Weber et al., 2009), and PI3K inhibitors stimulated *Legionella* infection also in macrophages (Peracino et al., 2010). Membrane phosphatidylinositides are important regulators of actin assembly/disassembly in the plasma membrane and in phago- or macropinosomes as well as phago- and macropinosome fusion with vesicles of the endo-lysosomal pathway. It was shown that altering phosphoinositide metabolism in *Legionella* infection resulted in even stronger inhibition of the *Legionella*-containing macropinosome with acidic vesicles, a process apparently stimulated by PI3P formation (Clarke et al., 2010; Peracino et al., 2010). This has led to the hypothesis that *Legionella* hinders fusion of the *Legionella*-containing macropinosome with acidic vesicles, but not vesicles bearing Nramp1, by inhibiting PI(3)P formation either by secreting a 3-P phosphatase or by anchoring a 3-P phosphatase of the host to the replication vacuole.

## The genetic basis of iron homeostasis in *Dictyostelium*

*Dictyostelium* cell growth is sensitive to iron depletion as well as to iron loading above 0.2 mM. When axenic wild type cells are incubated in minimal medium without iron, growth decreases dramatically and after 4–5 generations stops completely. *Nramp1* or *nramp2* knockout mutants fail to duplicate already after 2 generations. High iron concentrations reduce the growth rate in the wild type, to a lower extent in the *nramp* single knockout mutants, but only minimally in the double KO mutant. These results suggest that inactivating *nramp1* and *nramp2* leads to a lower intracellular level of bioavailable cellular iron, independently of the total amount that may enter the cell (Peracino et al., 2013). *Dictyostelium* development occurs under starving conditions in simple salt solutions, or even in water; addition of divalent transition metals is not required. During development, the only source of energy and organelle recycling is the autophagic breakdown of cellular components, which is responsible for the observed decrease in cell size (Kessin, 2001). Autophagy could also be the major source of iron during development, unless excess labile iron is accumulated during growth in the contractile vacuole, and the latter acts as iron reserve. It is worth mentioning that Nramp2 gene expression is stimulated by starvation, with maximal mRNA accumulation reached during aggregation and slug formation (Peracino et al., 2013).

In addition to the Nramp iron transporters, the *Dictyostelium* genome encodes many proteins involved in cellular iron homeostasis (Table 1). Homologs of the mitochondrial iron transporter mitoferrin (Satre et al., 2007), Fe-S and heme ABCB transporters (Anjard et al., 2002), and the iron sensor frataxin as well as a cytosolic and a mitochondrial aconitases are present. The cytosolic aconitase (Aco1) is highly similar to mammalian Iron Regulatory Protein (IRP), raising the possibility that it may bind iron regulatory elements (IREs), thus regulating iron-dependent genes (Anderson et al., 2012). Two distantly-related ferroportin (Slc40A1 or IREG1)-like proteins exist, but no homologs for trasferrin or transferrin receptors, the systemic iron traffic regulator hepcidin or the hepcidin inhibitor hemojuvelin are found, in agreement with the notion that iron is mainly derived from bacterial digestion. Ferritin or mitoferritin homologs are also not evident, though a putative ferritin-like protein, but with very low homology to other ferritin-like proteins, is encoded in the genome.

**Table 1:** **Selected “iron genes” in the *Dictyostelium* genome**.

**Gene product**	**Gene name**	**Dictybase ID**
Nramp1	*nramp1*	DDB_G0276973
Nramp2	*nramp2*	DDB_G0275815
Aconitase (cytosolic)	*aco1*	DDB_G0279159
Aconitase (mitochondrial)	*aco2*	DDB_G0278779
ABCB1	*abcB1*	DDB_G0293416
ABCB4	*abcB4*	DDB_G0279915
ABCB5	*abcb5*	DDB_G0292554
Mitoferrin	*mcfF*	DDB_G0269470
Frataxin	*fxn*	DDB_G0293246
*CytB561 ferric reductase*		DDB_G0279437
*CytB561 ferric reductase*		DDB_G0283271
*Slc40 family protein*		DDB_G0278675
*Slc40 family protein*		DDB_G0279065
*Ferritin-like superfamily protein*		DDB_G0278989

The listed ABCB transporters are homologs or orthologs of yeast and mammalian mitochondrial transporters (see Anjard et al., 2002). For mitoferrin see Satre et al., 2007. Genes in italics encode distantly-related proteins of the indicated families. For information on each gene see text and www.dictybase.org.

**Figure 2:**
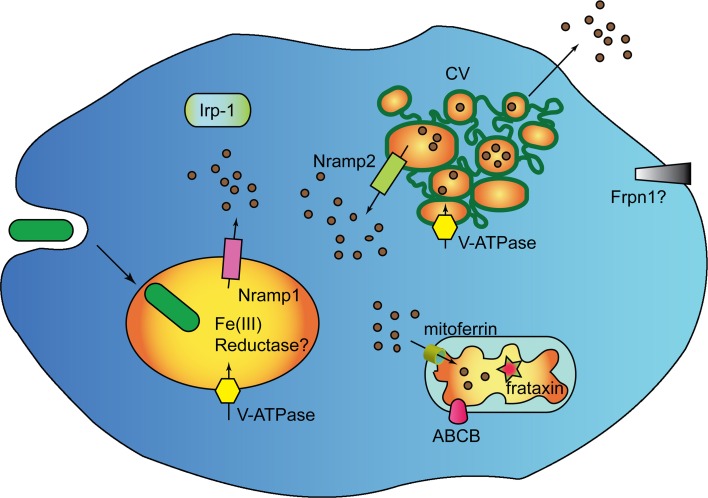
**Genes regulating iron homeostasis in *Dictyostelium***. The major, if not unique, source if iron for *Dictyostelium* cells are engulfed bacteria which are degraded in phago-lysosomes. The Nramp1 transporter is recruited to phagosomes shortly after uptake and is retrieved during post-lysosomal maturation. Nramp2 is localized in the membrane of the contractile vacuole (CV). It is assumed that Nramp2 acts like Nramp1, due to co-localization of the V-H^+^ ATPase in both phagosome and CV. The CV lumen is, however, not acidic, due to rapid buffering of the pumped protons, thus Nramp2 activity and directionality of transport could be modulated by other unknown factors. In addition to the Nramp transporters, the *Dictyostelium* genomes harbors homologs of the mitochondrial iron transporter mitoferrin, the iron sensor frataxin, and mitochondrial Fe-S and heme exporters of the ABCB family. In addition to mitochondrial ferrochelatase and aconitase *(not shown)*, the genome encodes a homolog of mammalian cytosolic aconitase IRP-1, which may act as iron-regulatory-protein by binding to iron-regulatory elements (IRE) in target proteins. Distant homologs of the membrane iron exporter ferroportin are also encoded in the genome, as well as two putative ferric reductases. It is not known whether any of the ferric reductases is located in phagosomes. *Dictyostelium* does not possess homologs of transferrin or transferrin receptors nor mitoferritin or ferritin, but a very distantly related ferritin-like protein. It is proposed that the CV acts as membrane-bound labile iron store, secreting excess iron by fusing with the plasma membrane or transporting iron back in the cytosol via Nramp2, when needed. In this model, the CV takes over ferritin, and possibly also ferroportin, function.

Bacterial digestion in phago-lysosomes will result in ferric ions that need to be reduced for transport via Nramp1 in the cytosol. In macrophages, where the major source of iron is represented by aged erythrocytes and bacteria, this is accomplished by ferric reductases of the STEAP family (Wang and Pantopoulos, 2011), which have no orthologs in *Dictyostelium* genome. Two putative ferric reductases of the domon-cytB561 family are, however, encoded in the genome, one of which is highly expressed during growth (Table 1). Whether any of them is localized in the phagosome, and may be responsible for ferric ion reduction, is under study. It is clear, in any case, that cells manage to reduce ferric ions, as ferric chloride added to minimal medium stimulates cell growth (Peracino et al., 2013). As summarized in the model in Figure 2, it can be hypothesized that following ferric ion reduction, iron is exported from the phagosomes via Nramp1 in a proton-gradient dependent co-transport. Most cytosolic iron will be transported to the mitochondria via mitoferrin, to be incorporated into Fe-S clusters and heme groups, a process likely regulated by frataxin, as in mammalian cells (Wang and Pantopoulos, 2011). Excess labile iron in the cytosol could accumulate in the contractile vacuole, either to be released following CV discharge upon fusion with the membrane or to be recycled to the cytosol via Nramp2, particularly under starving conditions, when the only source of iron is intracellular. In this model, the contractile vacuole is proposed to act as homeostatic vesicle-bound iron store, taking over ferritin function. In principle, also a plasma membrane iron exporter, such as ferroportin, would be dispensable in this model. If the model is correct, new questions arise: how is iron transport in the contractile vacuole regulated? Is Nramp2 the only iron transporter and does it mediate both import and export? How does the electrogenic potential generated by the V-H^+^ ATPase in the CV membrane regulate Nramp2 activity, taking in consideration that protons are efficiently buffered in the contractile vacuole lumen (Clarke and Heuser, 1997), contrary to what occurs for Nramp1 in the phago-lysosomes?

## Not only iron

In addition to iron, major divalent transitions metals that have been involved in host-pathogen interactions are manganese, copper, and zinc (Kehl-Fie and Skaar, 2010; Botella et al., 2012; Soldati and Neyrolles, 2012). Whereas iron and manganese are depleted from mature phagosomes, copper, and possibly zinc have been suggested to be pumped in to intoxicate bacteria; increased concentrations of both metals have been reported in *mycobacteria*l infection after stimulation of infected macrophages with cytokines (Wagner et al., 2006).

Very little is known about these metals in *Dictyostelium*. In contrast to iron, cell growth is unaffected by both manganese depletion or millimolar addition to minimal medium, and manganese does not rescue cell growth in iron-depleted medium (Peracino, unpublished results). Manganese can, however, stimulate cell aggregation and cell differentiation, as it directly stimulates the activity of adenylyl cyclase (Loomis et al., 1978; Hagmann, 1985) and of a bi-functional glycosyltransferase that regulates O_2_-dependent development (Trinchera and Bozzaro, 1996; West et al., 2010). *Dictyostelium* development is also sensitive to heavy metals present in the soil. Hg in particular inhibits development at concentrations of 50 mg per kg dry soil, compared to about 6-, 16-, and 32-fold higher concentrations for Fe, Zn, and Cu, respectively (Ponte et al., 2000; Balbo and Bozzaro, 2009; Rodriguez-Ruiz et al., 2013).

The *Dictyostelium* genome encodes several putative copper and zinc transporters. A Menkes type Cu^2+^ ATPase and a putative p80 copper transporter have been shown to be localized in the plasma membrane and in phagosomes. The unusual resistance to Cu has been linked to the high secretory efficiency of the Cu^2+^ ATPase (Burlando et al., 2002), which in the phagosome could pump Cu^2+^ ions in the lumen, favoring a potentially toxic effect of this metal on bacteria. A chemical assay for detection of metallothioneins, which could also account for copper resistance, failed to detect any activity, suggesting that *Dictyostelium* cells do not express these proteins (Burlando et al., 2002). The *Dictyostelium* genome harbors, however, one gene (DDB_G0288281), which has been annotated as putative metallothionein. Metallothioneins are small cysteine-rich proteins capable of binding heavy metals through the thiol group of their cysteine residues. Unfortunately, their primary structure is extremely variable and their secondary and tertiary structures are highly heterogeneous, making it difficult to recognize homologs among phyla, sometimes even among species (Coyle et al., 2002). Thus, whether the DDB_G0288281gene product is truly a metallothionein is open.

The p80 copper transporter is present in the plasma membrane and in endocytic vesicles. The protein is retrieved from the phagosome just after closure, but is then recruited again to mature phagosomes, probably by fusion with endocytic vesicles (Ravanel et al., 2001). In *M. marinum* infection, p80 has been shown to be recruited to the pathogen-replication vacuole at the onset of intravacuolar growth (Hagedorn and Soldati, 2007). Based on homology with other channel transporters, p80 is expected to transport copper outside of the vacuole, but no functional studies have been done, nor it is known whether disruption or overexpression of the gene affects host-pathogen interactions. The *Dictyostelium* zinc transporter family includes 12 members, most of which are expressed in late development and are assumed to be involved in cell differentiation (Sunaga et al., 2008). No data are available on potential localization of zinc transporters in phagosomes and their role during growth or host-pathogen interactions.

Recently, attention has been driven to a potential involvement of Mg^2+^ in nutritional immunity, based on results showing that synthesis of the *M. tuberculosis* virulence factor isoTb, which is involved in phagosome maturation arrest, was up- or down-regulated by Mg^2+^ overloading or depletion, respectively (Mann et al., 2011; Soldati and Neyrolles, 2012). In *Dictyostelium*, a V P-ATPase was identified as the tagged gene in the kil2 mutant, characterized by reduced phagosomal protease activity and defective growth on *Klebsiella pneumoniae* (Lelong et al., 2011). Addition of Mg^2+^ rescued the mutant, suggesting that the V P-ATPase could be a magnesium pump responsible for optimal Mg^2+^ concentration in the phagosome. Intriguingly, the kil2 mutant was able to grow on other bacteria species and did not display increased susceptibility to *M. marinum* infection.

## Conclusion

As obligate phagocytes, at least during the growth phase of their life cycle, *Dictyostelium* cells resemble macrophages for their ability to engulf bacteria and dead cells, to discriminate between self and non-self and to fight potential pathogens. They also share with macrophages several “iron genes” regulating cellular iron metabolism, though lacking genes involved in systemic iron homeostasis. The organism is thus a useful model for investigating iron homeostasis at cellular, rather than systemic, level and the role of iron (and other divalent metals) in host-pathogen interactions. The potential absence of ferritin, and possibly also ferroportin, homologs raises the question of how labile iron is neutralized and stored in the cell. The possibility that the contractile vacuole acts as iron (divalent metal ions?) store and/or sink, thus taking over the functions of ferritin and ferroportin, is very suggestive, opening novel potential roles for this compartment also in free-living amoebae and protozoa, but needs to be proven. Investigating the mechanism of action of Nramp2 can be of help in this context as well as developing more sensitive probes for detecting iron in intracellular compartments. The highly conserved function of Nramp1 in the phago-lysosomal membrane, from *Dictyostelium* to macrophages, corroborates the central role of iron control for the host and the pathogen in this compartment. The finding that Nramp2 KO mutants are sensitive to infection almost as much as Nramp1 KO mutants raises the question of potential cross-talk between the phago-lysosome (or the pathogen-replication vacuole) and the contractile vacuole in iron homeostasis and in resistance to pathogens. The ease in generating and analysing mutants in *Dictyostelium* will help in the near future in dissecting the role of mitochondrial and cytosolic iron genes in these intracellular interactions and in extending these studies to genes regulating metabolism of other divalent metals relevant for host-pathogen interactions.

### Conflict of interest statement

The authors declare that the research was conducted in the absence of any commercial or financial relationships that could be construed as a potential conflict of interest.
